# Management of Obese Type 1 Diabetes Mellitus (Double Diabetes) Through Telemedicine During COVID-19 Pandemic Lockdown: A Case Report

**DOI:** 10.7759/cureus.30533

**Published:** 2022-10-21

**Authors:** Swar Gupta, Sangita Totade, Kavita Gupta, Parvinder Bamrah, Shlok Gupta, Sunil Gupta

**Affiliations:** 1 Pharmacology, Jawaharlal Nehru Medical College, Wardha, IND; 2 Nutrition, Sunil’s Diabetes Care n’ Research Centre, Nagpur, IND; 3 Nutrition, Sunil's Diabetes Care n' Research Centre, Nagpur, IND; 4 Internal Medicine, Sunil's Diabetes Care n' Research Centre, Nagpur, IND; 5 Endocrinology, Sunil's Diabetes Care n' Research Centre, Nagpur, IND

**Keywords:** weight loss and obesity, national lockdown, previous gestational diabetes mellitus, type 1 diabetes mellitus (t1d), covid-19 telemedicine, medical nutrition therapy, double diabetes, covid-19 retro

## Abstract

Metabolic syndrome in Type 1 diabetes mellitus (T1DM) has been shown to be an independent risk factor for macro-vascular and micro-vascular complications. Obesity also affects many people with T1DM across their lifetime with an increasing prevalence in recent decades. Individuals with T1DM who are overweight, have a family history of type 2 diabetes, and/or have clinical features of insulin resistance, are known as "double diabetes". It is challenging for a person with double diabetes to achieve reasonable glycemic control, avoid insulin-related weight gain, and prevent hypoglycaemia. This was especially true during the coronavirus disease 2019 (COVID-19) pandemic lockdown. The aim of this report is to show that lifestyle modification through telemedicine can immensely help in managing uncontrolled T1DM with associated morbid obesity in lockdown situations, with the help of the diabetes educator. In this case, the complicated history of double diabetes was taken through telephonic and online consultations with the help of a nutritionist and diabetes educator, and the treating clinician supervised the insulin doses and frequency. Patient Health Questionnaire (PHQ)-9 questionnaire was used to assess depression. Medical nutrition therapy (MNT) was given through online consultations, where the patient was reoriented to carbohydrate counting, insulin dose adjustment, along with modifications in the diet. Regular exercise was advised along with frequent self-monitoring of blood glucose (SMBG). Moreover, the diet order was changed to eat protein and fibre first, followed by carbohydrates, later. The three-tier system of the medical expert, clinical dietitian, and diabetes educator was applied. The subject was trained for carbohydrate counting and insulin dose adjustment by teaching her about the insulin-to-carb ratio and insulin sensitivity factor (ISF). She was asked to examine her insulin injection sites by visual and palpatory methods for lipohypertrophy. Once a week, the diabetes educator and nutritionist did telephonic follow-up and counselling, while online consultation was done by the treating clinician once a month. As a result, her weight, BMI, and waist circumference were reduced drastically, and there was an improvement in haemoglobin A1C (HbA1C), lipid parameters, and blood pressure after the intervention. Thus, implementing diabetes education via telemedicine in circumstances such as the COVID-19 pandemic can help achieve the best possible compliance for strict diet adherence, regular exercise and monitoring, reducing obesity, glycosylated HbA1c, insulin doses, and risk of depression in a person with double diabetes.

## Introduction

A person with type 1 diabetes mellitus (T1DM) is traditionally described as lean and insulin-sensitive, where insulin deficiency rather than insulin resistance is the primary pathophysiological mechanism. The global increase in overweight and obesity, the so-called obesity epidemic [1], is associated with metabolic disturbances like insulin resistance, hyperinsulinemia, dyslipidemia, and subclinical inflammation, which results in the development of micro and macro-vascular diseases [2].

Obesity also affects a large number of people with T1DM across their lifetime, with an increasing prevalence in recent decades and with rates ranging from 2.8% to 37.1% [3], which is termed "double diabetes" [4]. Insulin resistance and tight glycemic control also increase weight, insulin demand, and the risk of hypoglycemia [5]. A sedentary lifestyle, a high-calorie diet rich in fats and simple sugars, and a low-fibre diet in T1DM also lead to poor metabolic control, weight gain, and affective disorders like depression that further aggravate the condition [6]. Metabolic syndrome in T1DM has been shown to be an independent risk factor for macro-vascular and micro-vascular complications [7].

Managing uncontrolled T1DM on a high insulin dose with associated morbid obesity is challenging and may also be accompanied by behavioural changes like eating disorders or associated depression, especially during situations such as the coronavirus disease 2019 (COVID-19) pandemic lockdown, which may further complicate the diabetes management. Studies have used Diabetes Eating Problem Survey (DEPS-R) for the diagnosis of eating disorders and the Patient Health Questionnaire (PHQ)-9 scale for the assessment of depression [8,9]. Lifestyle modification through telemedicine can play a vital role in such cases, which, if effectively implemented, can give rewarding results.

## Case presentation

Patient information

A 33-year-old female homemaker, vegetarian, with a diagnosis of T1DM for 24 years, currently staying at her hometown, presented with uncontrolled diabetes, despite high doses of insulin, increasing weight, limitation of movements, tiredness, exhaustion, and emotional instability with negative thoughts. She was on subcutaneous insulin basal-bolus therapy and had a sedentary lifestyle due to the COVID-19 lockdown. She has polycystic ovarian syndrome (PCOS), had pre-gestational diabetes mellitus nine years ago, and delivered a normal baby through a lower (uterine) segment Caesarean section, without significant perinatal complications.

The patient was also hypertensive for four years, which was well controlled on telmisartan 20 mg once a day. She had non-proliferative diabetic retinopathy, and there was no other significant past, family, or personal history. She was on basal-bolus insulin therapy in divided doses, 140 units/day. She was also on metformin for polycystic ovarian syndrome (PCOS) and voglibose for postprandial hyperglycaemia. She was leading a sedentary lifestyle with minimal activity since the lockdown in March 2020, that is for the past six months till the presentation.

Clinical picture

She consulted us via teleconsultation from a distant city in September 2020. For the past seven years, she had been managing her blood glucose levels by herself and had not consulted anyone else during this period. She was performing the SMBG testing method using the glucometer and was not using the continuous glucose monitoring (CGM) device. Her height, weight, BMI, waist circumference, and haemoglobin A1C (HbA1C) were 150 cm, 67.8 kg, 30.1kg/m^2^, 101 cms, and 8.7%, respectively. The blood pressure measurement was taken at home by herself using an electronic blood pressure apparatus, of a standard cuff size of 16 cm by 36 cm. Her mid-arm circumference was 35 cm. Also, there was a gradual increase in weight in the past three years. Other biochemical parameters were within physiological limits.

She was on the following treatment before intervention: Inj. human regular insulin: 34IU before breakfast (BBF), 33IU before lunch (BL), 33IU before dinner (BD); Inj. human neutral protamine Hagedorn (NPH) insulin: 20IU twice daily (at 9 am and 9 pm): Total daily dose (TDD) of insulin: 140 units/day; tab. metformin 1000 mg twice daily (for PCOS); tab. voglibose 0.3 mg twice daily (for postprandial hyperglycaemia); tab. telmisartan 20 mg once daily at dinner (for hypertension).

Diagnostic assessment

Her history was taken through telephonic and online consultations. The important parameters of history like easy fatiguability, nocturnal micturition, pre-gestational diabetes mellitus, and PCOS were recorded. The clinician, nutritionist, and diabetes educator engaged both in the conference calls with the patient and her husband and also without the patient to formulate further treatment protocol, so as to improve the condition of the patient. The PHQ9 questionnaire was used to assess depression.

The initial PHQ9 score was 10, which depicts that she was suffering from moderate depression. The DEPS-R scale showed that she did not have any eating disorder. The treating clinician supervised the insulin doses and frequency. Details like the presenting complaints, history of presenting illness, treatment history, 72 hours of dietary recall with food frequency, physical activity profile, and history of associated complications were reviewed online by the nutritionist and diabetes educator.

Therapeutic interventions

Medical nutrition therapy (MNT) was carried out through online consultations. Many T1DMs may underestimate their calorie consumption. However, the patient was literate and was using the kitchen weighing scale. She was given training in food exchange and the hand & plate method. Past energy intake was 1700-1800 kcals/day (carbohydrate 259 g, protein 46 g, fat 54 g), calculated by the 24-hour dietary-recall method by the nutritionist. The food frequency questionnaire revealed that fast/fried food and bakery food consumption were twice and three to four times a week, respectively, while nuts, fruits, and green leafy vegetables were consumed once a week. Her total calorie intake was reduced to 1200 kcals/day (carbohydrate 195 g, protein 72 g, fat 36 g), including a moderate carbohydrate, low fat, high fibre diet, and introducing free foods (containing less than 20 calories or 5 g carbohydrate per serving). The patient was aware of carbohydrate counting, insulin dose adjustment, insulin-to-carb ratio (ICR) (ICR=450/total daily dose (TDD)), and insulin sensitivity factor (ISF) 1700/ TDD. However, she was not compliant and wasn't following these methods. Hence, she was counselled about their importance.

Regular exercise of 45-60 mins (walking, jogging) in two to three sessions was advised, along with a more frequent SMBG. Postprandial blood glucose was high, and hence, protein snacks replaced carbs. The food order was changed to eat protein and fibre first, followed by carbs later. The diabetes educator played an essential role in getting optimum diet, lifestyle changes, blood glucose monitoring compliance, etc., through multiple telephonic calls, WhatsApp (Meta Platforms, Inc.Menlo Park, California, United States), and frequent online meetings. The three-tier system of the medical expert, clinical dietitian, and diabetes educator was applied. The timeline for the intervention was around 16 weeks or 120 days. She was asked to examine her insulin injection sites by visual and palpatory methods for lipohypertrophy. Once a week, the diabetes educator and nutritionist did telephonic follow-up and counselling, while online consultation was done by the treating clinician once a month.

Follow-up and outcome of the intervention

Five months after the intervention, improvements in lipid parameters and blood pressure were seen. Except for insulin doses, other medications remained unchanged. Her PHQ9 score dropped down to 5, indicative of decreased severity of depression. The basal-bolus dose of insulin after the intervention was: Inj. human actrapid (regular) insulin 06IU-18IU-14IU (BBF, BL, and BD, respectively); Inj. human insulatard (NPH) insulin 06IU at 9 am and 15IU at 9 pm: TDD of insulin 59 units/day.

Her weight, BMI, and waist circumference were reduced by 10%, 10%, and 14.85%, respectively. The TDD of insulin was reduced by 56%, while her HbA1C, fasting, and post-meal blood glucose were reduced by 11.5%, 20%, and 29%, respectively, from baseline (Table 1).

**Table 1:** Changes in the Blood Pressure, Anthropometric and Biochemical Parameters BMI: Body Mass Index; FBG: Fasting Blood Glucose Test; PPBG: Post-Prandial Blood Glucose; eGFR: Estimated Glomerular Filtration Rate Test; SGOT: Serum Glutamic-Oxaloacetic Transaminase Enzyme Test; SGPT: Serum Glutamic Pyruvic Transaminase Enzyme Test; LDL: Low-Density Lipoprotein; HDL: High-Density Lipoprotein

	Sept 2020 (Baseline)	January 2021 (Post-intervention)	Post-intervention % Change
Weight (kgs)	67.8	61	Decreased by 10%
BMI (kg/m2)	30.1	27.1	Decreased by 10%
Waist (cms)	101	86	Decreased by 14.85%
Insulin Total Daily Dose (units/day)	134	59	Decreased by 56%
FBG (mg/dl)	200	160	Decreased by 20%
PPBG (mg/dl)	240	170	Decreased b 29%
HbA1C (%)	8.7	7.7	Decreased by 11.5%
Cholesterol (mg/dl)	186	143	Decreased by 23.1%
Triglyceride (mg/dl)	156	120	Decreased by 23%
LDL Cholesterol (mg/dl)	116	77	Decreased by 33.6%
HDL Cholesterol (mg/dl)	38.8	42	Increased by 8.3%
Sr. Creatinine (mg/dl)	0.5	0.5	NIL
eGFR	123	123	NIL
SGPT (mg/dl)	22	23	NIL
SGOT (mg/dl)	21	22	NIL
Uric Acid (mg/dl)	3.45	3.50	NIL
Microalbumin (mg/dl)	28	23	NIL
Systolic Blood Pressure (mm of Hg)	140	130	Decreased by 7.1%
Diastolic Blood Pressure (mm of Hg)	90	82	Decreased by 8.9%

## Discussion

The prevalence of obesity is increasing globally, which not only increases the risk of type 2 diabetes mellitus but also affects people with T1DM, primarily due to changing dietary habits and poor exercise compliance. Management of such cases of double diabetes is challenging.

On the evening of March 24, 2020, the Government of India ordered a nationwide lockdown to limit the movement of India's entire 1.38 billion (138 crores) population as a preventive measure against the COVID-19 pandemic in India [10]. People with uncontrolled diabetes are believed to have a higher risk of complications, severity, and death. Studies have shown the potential benefits of remote telemedicine in diabetes care, and its use is rapidly increasing due to the pandemic [11-13]. In a systematic review of 29 studies in paediatric diabetes care, it was concluded that telemedicine has the potential to facilitate patient monitoring and improve short-term glycemic control in some contexts [14]. 

While discussions about remote telemedicine are rapidly increasing due to the pandemic, a number of studies have previously investigated the potential benefits of telemedicine in diabetes care [15]. A recently published perspective article highlighted how paediatric patients with T1DM have historically led the way in the adoption of diabetes technology [16]. In their article, Danne and Limbert identify how young patients have been particularly receptive to new technologies such as insulin pumps and glucose sensors, suggesting the trend would continue for telemedicine [16].

This strategy of telemedicine approach is unique because it is usually just the clinician and dietitian who are involved in the treatment protocol. This may decrease the efficiency of the treatment, as most of the time, patients may not be compliant enough to follow all the prescribed medications and techniques regularly, and it may not be possible to take regular follow-ups by healthcare professionals. Hence, diabetes educators can proactively get involved, play an important role as the connecting link, and collectively help in increasing the effectiveness of the treatment, achieving the desired goals at a faster pace.

Weight management is a continuous process, and the patient has been counselled for consistent regular follow-ups with the team regarding the same, targeting HbA1c between 6.5-7%, and BMI less than 23kg/m^2^, which is the cut-off for Asians. Urine spot microalbumin creatinine ratio must be checked as it is an independent risk factor for cardiovascular diseases. Once there is a further decrease in weight, the insulin doses will be consequently reduced, and we shall also withdraw the oral drug, voglibose in the subsequent follow-ups. We shall also perform annual screening for target organ damage to assess any structural or functional impairment of major body organs. Thus, our case report has shown that telemedicine through structured virtual/telephonic connections can also help patients improve their condition, even when they cannot avail themselves of facilities from faraway places, especially in a challenging case like double diabetes (obese T1DM).

## Conclusions

Through a holistic approach, diabetes education can be implemented via telemedicine for lifestyle modification by a diabetes educator and nutritionist under a clinician's guidance. This can help us to achieve the best possible compliance for strict diet adherence, regular exercise, and monitoring. Moreover, it is also evident from our case report, that people with T1DM can be managed in a very efficient manner, if they are well acquainted with the technology and can achieve the possible glycemic targets, even if they are situated in remote areas.

Telemedicine can help in weight control, reducing glycosylated HbA1c, lowering blood pressure, achieving better glycemic control on lesser insulin doses, and improving lipid parameters. It also offers a better quality of life by reducing the risk of depression in a person with double diabetes (obese T1DM) during times like the COVID-19 pandemic when in-person consultation is not possible.
